# Distribution of Bottom Trawling Effort in the Yellow Sea and East China Sea

**DOI:** 10.1371/journal.pone.0166640

**Published:** 2016-11-17

**Authors:** Shengmao Zhang, Shaofei Jin, Heng Zhang, Wei Fan, Fenghua Tang, Shenglong Yang

**Affiliations:** 1 Key Laboratory of East China Sea & Oceanic Fishery Resources Exploitation and Utilization, Ministry of Agriculture, P.R. China, East China Sea Fisheries Research Institute, Chinese Academy of Fishery Sciences, Shanghai, China; 2 Key Laboratory of Wetland Ecology and Environment, Northeast Institute of Geography and Agroecology, Chinese Academy of Sciences, Changchun, China; 3 Key Laboratory of Geographic Information Science, Ministry of Education, East China Normal University, Shanghai, China; Bangor University, UNITED KINGDOM

## Abstract

Bottom trawling is one of the most efficient fishing activities, but serious and persistent ecological issues have been observed by fishers, scientists and fishery managers. Although China has applied the Beidou fishing vessel position monitoring system (VMS) to manage trawlers since 2006, little is known regarding the impacts of trawling on the sea bottom environments. In this study, continuous VMS data of the 1403 single-rig otter trawlers registered in the Xiangshan Port, 3.9% of the total trawlers in China, were used to map the trawling effort in 2013. We used the accumulated distance (AD), accumulated power distance (APD), and trawling intensity as indexes to express the trawling efforts in the Yellow Sea (YS) and East China Sea (ECS). Our results show that all three indexes had similar patterns in the YS and ECS, and indicated a higher fishing effort of fishing grounds that were near the port. On average, the seabed was trawled 0.73 times in 2013 over the entire fishing region, and 51.38% of the total fishing grounds were with no fishing activities. Because of VMS data from only a small proportion of Chinese trawlers was calculated fishing intensity, more VMS data is required to illustrate the overall trawling effort in China seas. Our results enable fishery managers to identify the distribution of bottom trawling activities in the YS and ECS, and hence to make effective fishery policy.

## Introduction

Bottom trawling is a high-efficiency fishing technique in global coastal fisheries [1], but with negative effects on marine benthos [2]. Bottom trawling affects seabed environments by dragging a net on the seabed and suspending sediments [3]. In addition, this fishing activity threatens marine benthic biodiversity and alters the structures of benthic ecosystems [4]. Fishers have been concerned with these impacts for 600 years [5]. Over the past half century, the impacts of bottom trawling have come to the forefront for scientists and governments [5, 6]. To reduce the negative effects on benthic ecosystems from bottom trawling, the European Union has used a Vessel Monitoring System (VMS) to monitor the activities of all vessels greater than or equal to 24 m long since 2000 and 15 m long since 2005 [7]. VMS has been shown to be an effective surveillance and enforcement tool for monitoring spatial and temporal fishing distributions [8]. Furthermore, it is helpful to quantify the effects of fishing on various ecosystem components to inform ecosystem-based management [9].

China plays significant roles in the world’s marine fishery [10]. According to the China Statistical Yearbook of 2013, more than six million tons of catch were generated by China’s 36,744 trawlers [11]. The Chinese Ministry of Agriculture began to invest in the construction of the Beidou fishing vessel position monitoring system in 2006. At the end of 2014, more than 60 thousand fishing vessels had been outfitted with VMS. This system provides continuous records to support fishery management. Sixty percent of the trawlers in China are fishing in the Yellow Sea and the East China Sea. The Yellow Sea (YS) is a semi-closed area of the Northwest Pacific Ocean with an average depth of 44 m. The East China Sea (ECS) lies over the broad shelf of the Northwest Pacific Ocean with an average depth of 370 m. Trawling for Chinese fishery in the two seas mainly occurs in seawater at a depth to 300 meters. Based on our *in situ* investigation, the common warp length of trawlers in the YS and the ECS is several times longer than the seawater depth; thus, warps and nets inevitably plow the seabed when fishing. Consequently, these fishing activities can directly affect marine benthic environments. Moreover, several tows are done by a trawler during a single fishing trip. To investigate the impacts on these seas, the primary issue is to identify which regions are heavily affected by trawling. Few studies on this subject have been carried out in the China Seas, although several studies have applied VMS data to trace fishing activities in the China Seas [12–15].

Therefore, to address this issue and expand our knowledge of the impacts of trawling on the YS and ECS, the accumulated distance (AD), accumulated power distance (APD), and trawling intensity were calculated as indexes to detect heavily trawled regions. The AD is a distance index that is calculated by time and speed and represents the total ploughing distance on the seabed. The APD is a power index that is calculated by AD and engine power, and represents the total fishing cost. The trawling intensity is the ratio of trawled area to the total area and represent the trawling efforts. Furthermore, the final aim of this study is to provide new information for policy-makers charged with governing trawls.

## Materials and Methods

### Fishing vessel position data

Fishing vessel position data were provided by the management service provider of the China Beidou System. These data contained positions, time, speed, and other geographic information for each vessel registered by the Chinese Fishery Bureau. All data had a temporal resolution of three minutes and a spatial resolution of ten meters.

### Calculation of indicators

The AD and APD were calculated using Eqs 1 and 2, respectively. The total AD and APD for the YS and the ECS was summed over each 0.01°× 0.01° grid in the sea as follows:

$$AD=\sum_{i=0}^{m}\sum_{j=0}^{n}\sum_{k=0}^{p}\left(\frac{V_{i,j,k}+V_{i,j,k-1}}{2}\right)\times \left(t_{i,j,k}-t_{i,j,k-1}\right)$$ (1)

$$APD=\sum_{i=0}^{m}\sum_{j=0}^{n}\sum_{k=0}^{p}\left(\frac{V_{i,j,k}+V_{i,j,k-1}}{2}\right)\times \left(t_{i,j,k}-t_{i,j,k-1}\right)\times W_{i}$$ (2)

where *m* is the number of total trawler vessels, *n* is the number of total fishing nets, *p* is the number of spatial cells in the seas, *V* is the speed of vessel, *t* is the fishing time, and *W*_*i*_ is the power of the vessel. When a given distance in one time interval (3 min) fall in adjacent cells, the distance were considered as the first cell.

The bottom trawling intensity was stated as swept area ratio [16, 17]. It was the ratio of the area trawled to the total area in each cell. The swept area in one cell was expressed as Eq 3:

$$Swept\;area=\sum_{i=0}^{m}AD_{i}\times {\left(gear\;width\right)}_{i}$$ (3)

where *m* is the number of total trawler vessels in one cell, *AD*_*i*_ is the AD (km) for the *i* vessel, the *(gear width)*_*i*_ is the gear width (m) for the *i* vessel.

Bottom trawling vessels registered in this study are mainly single rig otter trawler, and operate the benthic fauna, including fish (e.g. *Trichiurus japonica*, *Larimichthys polyactis*), crustacean (e.g. *Metapenaeopis lata*), and cephalopod (e.g. *Loligo kobiensi*). In 2013, for the Xiangshan Port chosen in this study, there were 3139 fishing vessels, including 1403 bottom otter trawling, and the total landing was 0.44 million tons (fish: 0.34 million tons; crustacean: 58.40 thousand tons).

In addition, vessels with different engine power have different gear width. We collected the power data and their corresponding gear width in Chinese trawlers (See S1 File for the raw data). Furthermore, we gave the best fit function between the power and gear width for China trawlers. The functions were fitted using four types: linear, logarithmic, power, and saturating. The function with lowest Akaike Information Criterion (AIC) is expressed as the best fitted model (Table 1). For trawlers in China, the best relationship between power and gear width is expressed as Eq 4:

$$gear\;width={8.8576\times power}^{0.398}$$ (4)

where *gear width* is the width (m) of trawler, *power* is the engine power (kW).

**Table 1:** Results from Akaike Information Criterion (AIC) analyses for the four type functions between the gear width and power.

Function	AIC	AIC weight	Delta AIC
Linear	373.86	0.31	1.29
Logarithmic	376.27	0.09	3.70
**Power**	**372.57**	**0.59**	**0.00**
Saturating	384.73	0.00	12.16

Bold value highlight the best-fitting model.

### Registered Trawlers in the Xiangshan Port

**Fig 1:**
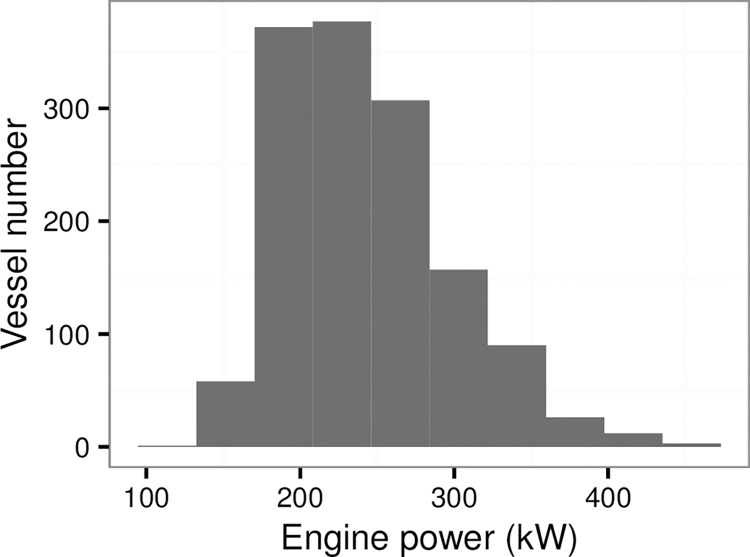
Frequency of the engine power of the otter bottom trawlers in the Xiangshan Port.

Due to different VMS operators in China, the VMS data for all Chinese bottom trawlers are unavailable at current stage. Therefore, in this study, we chose 1403 registered trawler vessels to address the AD and APD in the Xiangshan Port, which is one of the largest fishing ports in the ECS and has monitored all fishing all vessels. [10]. Among registered bottom trawlers, engine power ranges from 50 to 450 kW (Fig 1).

### Determination of towing speed threshold

**Fig 2:**
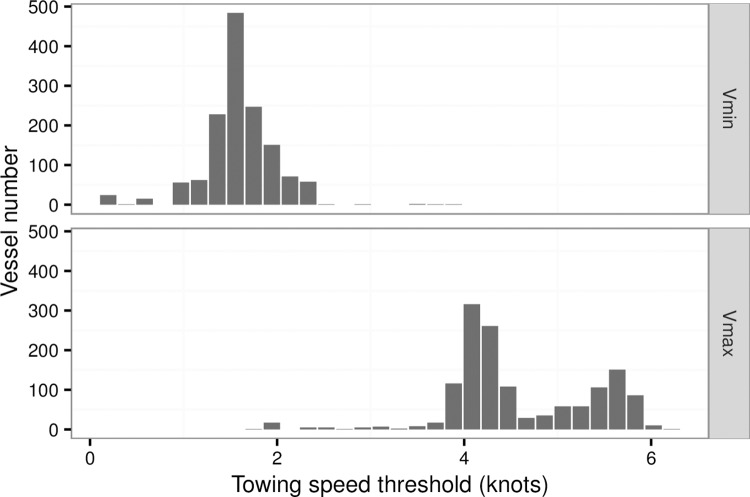
Frequency of towing speed threshold (minimum speed [Vmin] and maximum speed [Vmax] of towing) for the otter bottom trawlers in the Xiangshan Port.

Given previous studies [18–22], towing speed ranged between 2 knots to 6 knots, while, for an individual vessels, different engine power vessels correspond with different towing speed ranges. To determine the fishing activities accurately, we inquired about the towing speed with the skippers and collected the parts of observer notes of the all 1403 vessels in this study. According to these records, the minimum and maximum speed of towing were extracted for each vessel (Fig 2). Thus, two states, fishing and not-fishing activities, were determined as follows Eq 5:

$$P=\left\{ \begin{array}{l} \;non-fishing\;v\in [0,V_{min})\\ \;fishing\;v\in [V_{min},V_{max}]\\ \;non-fishing\;v\in (V_{max},+\infty ) \end{array}\right.$$ (5)

where *V*_*min*_ and *V*_*max*_ are the minimum and maximum towing speeds from the two states, respectively. The towing speeds ranged from 1.5 knots to 6.0 knots for the 1403 vessels in this study (Fig 2). Due to different engine power, the maximum speed threshold was generally between 1.7 knots and 6.2 knots (Fig 2).

### Brief introduction of fishing grounds in the East China Sea and Yellow Sea

**Fig 3:**
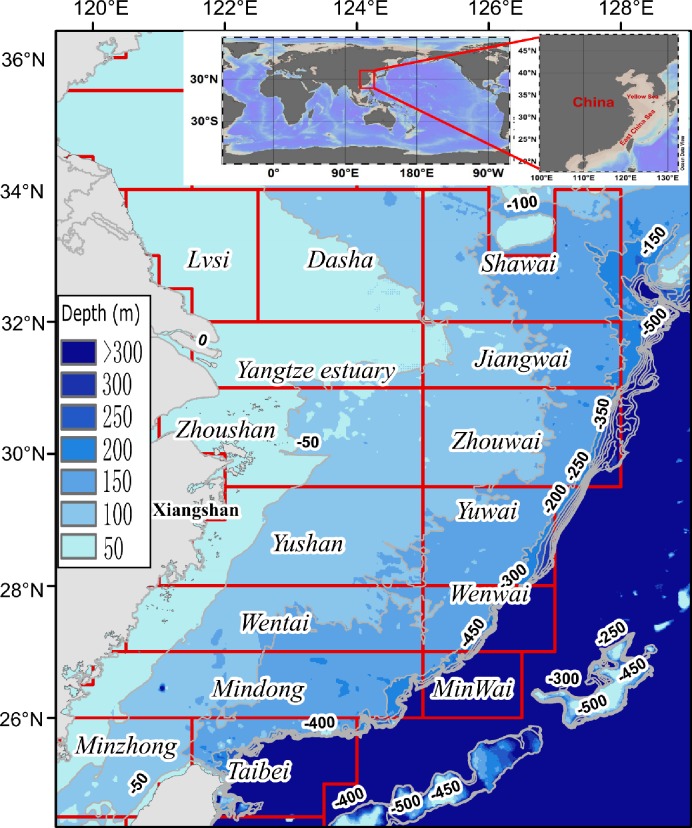
Distribution of fishing grounds in the Yellow Sea and East China Sea. Red polygons represent different fishing grounds.

Fig 3 showed the distribution of fishing grounds in the ECS and YS. Chinese fishermen had utilized the fishing resources in these grounds over hundred years. To date, these fishing grounds still play significant roles in the coastal fishery of China. Interestingly, the fishing grounds had been defined by ancient fishermen, and their names mainly came from the names of places, mountains, or rivers nearby.

### Limitations

Although all registered trawlers in the Xiangshan Port were collected in this study, the coverage of this study was still relative low. The indicators here represent the fishing intensity of trawlers in the Xiangshan Port. Based on the fishery yearbook in 2013 [11], China has a total of 36744 trawlers, which include all type of trawlers. The registered single rig otter trawlers in the Xiangshan Port only account for 3.9% of the total trawlers in China. The total marine landing in 2013 in China was 12.6 million tons, and the trawler fishery accounts for 52.23% of the total marine landing of China. The landing of the bottom trawlers in this study accounts for 6.05% of the total landing in China. Therefore, one must be noted that the trawling intensity were probably underestimated for the total fishing grounds in the ECS and YS. In addition, distance from port to fishing grounds plays critical roles in fishing activities for fishermen. The results in this study are most complete for fishing grounds around the Xiangshan Port (mainly Zhoushan, Yushan, Zhouwai, and Yuwai, Fig 3).

## Results

### Distribution of accumulated distance and accumulated power distance in different fishing grounds in the Yellow Sea and East China Sea

**Fig 4:**
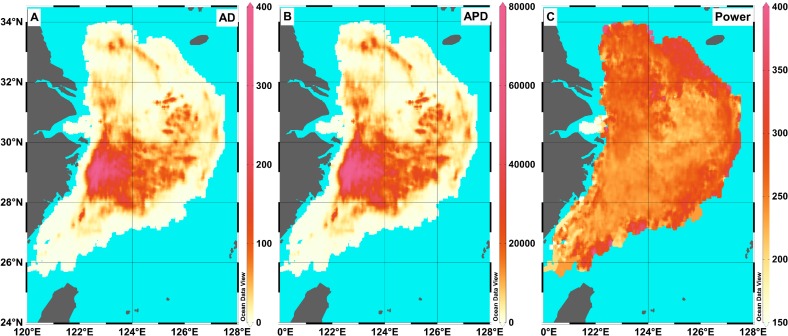
Distribution of (A) accumulated distance (AD, in km) and (B) accumulated power distance (APD, in km kW) of trawl vessels, and distribution of (C) the mean engine power (in kW) in the Yellow Sea and East China Sea in 2013.

The AD and APD were calculated in each 0.01° ×0.01° grid cell (See the S2 File for the raw data). The maximum AD values were found in the Yushan fishing ground and south of the Zhoushan fishing ground (Fig 4A), which is near the Xiangshan Port. In addition, the Zhouwai and Yuwai fishing grounds had higher AD values compared to other fishing grounds (Table 2). The APD had similar distribution pattern with the AD (Fig 4B). The greatest APD values were mainly located in four fishing grounds: the Zhoushan, the Yushan, the Zhouwai, and the Yuwai (Fig 4B and Table 2). Furthermore, bottom trawlers with greater engine power fishing further to the port (Fig 4C).

**Table 2:** List of trawled cells, fraction of no fishing area, accumulated distance (AD), accumulated power distance (APD), and trawling intensity for each fishing ground in the Yellow Sea and East China Sea in 2013.

Fishing ground	Total cells^a^	Trawled cells	Non-trawled cells	Non-trawled area ratio (%)	AD (×10^4^km)	APD (×10^6^) km·kW	Intensity (year^-1^)
Yushan	52500	44262	8238	15.69	597.82	1421.1	3.71
Zhoushan	55000	37307	17693	32.17	213.03	521.67	1.28
Yuwai	30000	24120	5880	19.60	92.13	238.71	1.04
Zhouwai	45000	35953	9047	20.10	130.17	314.27	0.95
Wentai	45000	28915	16085	35.74	71.96	171.36	0.52
Yangtze estuary	35000	22615	12385	35.39	46.73	123.63	0.45
Jiangwai	30000	19012	10988	36.63	38.38	92.97	0.42
Dasha	50000	35761	14239	28.48	60.39	162.27	0.41
Shawai	50000	11638	38362	76.72	8.05	24.94	0.05
Mindong	52500	10374	42126	80.24	4.43	10.41	0.03
Wenwai	20000	3124	16876	84.38	1.02	2.92	0.02
Lvsi	32500	1917	30583	94.10	1.2	3.22	0.01
Minzhong	35000	692	34308	98.02	0.08	0.19	0.00
Taibei	35000	246	34754	99.30	0.05	0.14	0.00
Total	567500	275936	291564	51.38	1265.54	3087.81	0.73

^a^: cell number at 0.01° ×0.01°.

### Trawling intensity in the fishing grounds

**Fig 5:**
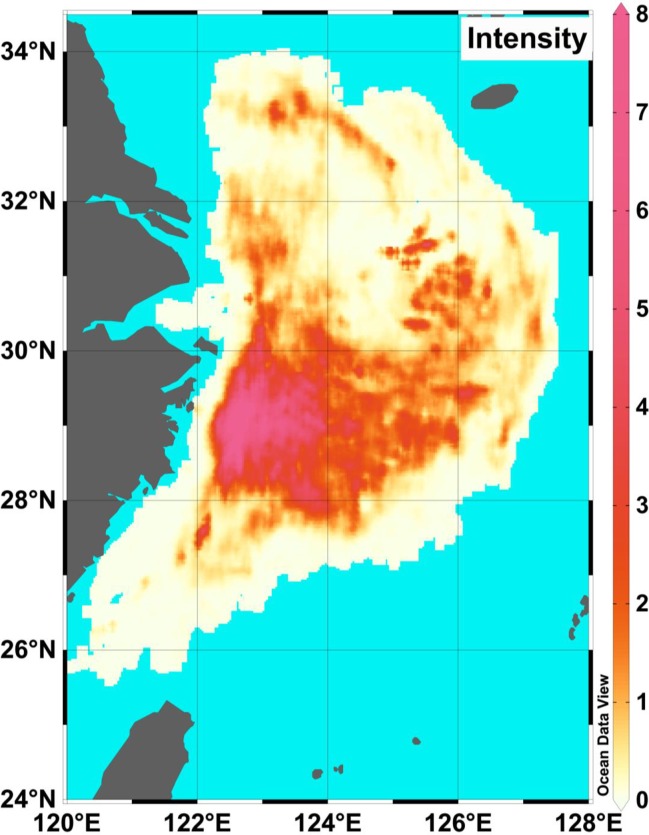
Distribution of trawling intensity (y^-1^) in the East Sea and Yellow Sea

**Fig 6:**
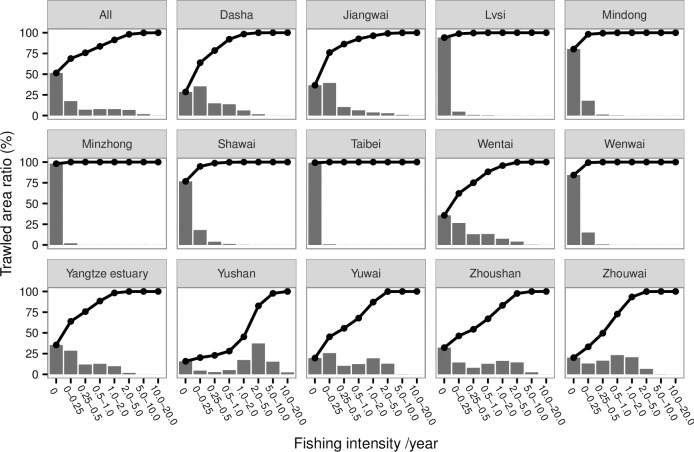
Trawled area ratio and cumulative trawled area ratio in total fishing grounds and each fishing ground. The bars represent the trawled area ration at different intensity categories. The lines represent the cumulative trawled area ratio.

Table 2 shows the total number of grid cells, trawled number of grid cells, non-trawled number of grid cells, AD, APD, and trawling intensity in each cell for the different fishing grounds in the YS and ECS (See the S2 File for the raw data). The two nearest fishing grounds (Yushan and Zhoushan) to the Xiangshan Port had greater mean AD and APD than other grounds. The Yushan and Zhoushan fishing grounds accounted for 64.07% and 62.91% of the total fishing AD and APD, respectively. Fishing grounds with greater AD had greater trawling intensity (Table 2). Further, Table 2 also ranked the fishing intensity in different fishing grounds. The fishing intensity in the three fishing grounds, Yushan, Zhoushan, and Zhouwai, were greater than one in 2013 (Table 2). The mean trawling intensity was 3.71 year ^-1^, 1.28 year ^-1^_,_ and 1.04 year^-1^ for the fishing grounds of Yushan, Zhoushan and Zhouwai fishing, respectively (Table 2). The fishing grounds near the port were more intensively trawled (Fig 5). The trawling intensity in the YS and ECS was 0.73 year^-1^ (Table 2). Further, no fishing occurred in 51.38% of grid cells on the fishing grounds (Table 2 and Fig 6). The trawling intensity in the 32.13% of the total area was between 0 and 1 (Fig 6). Fig 6 also showed the fraction of trawled area and cumulative trawled area for each fishing ground. Among the fishing grounds, the Yushan fishing ground had different cumulative pattern from others, where the trawled area with fishing intensity less than one accounted for the largest fraction of total area (Fig 6). However, in the Yushan fishing ground, the fishing area with intensity between two and ten accounts for 69.75% of the total fishing area.

### Monthly trends of accumulated distance and accumulated power distance

**Fig 7:**
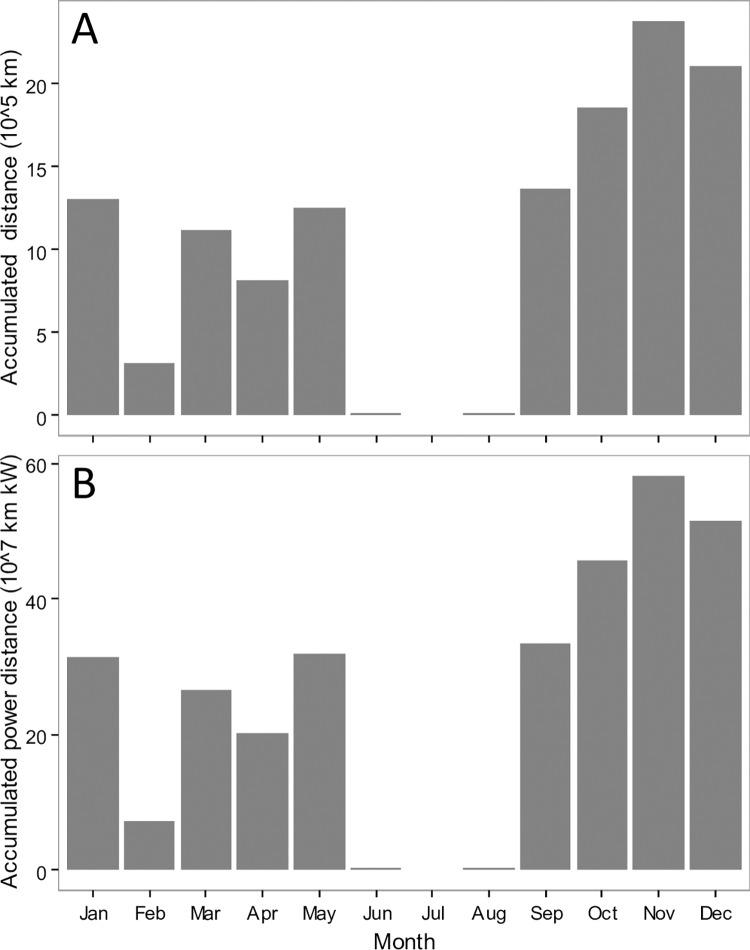
Monthly distribution of accumulated distance (A) and accumulated power distance (B) in the Yellow Sea and East China Sea in 2013.

Bottom trawlers was banned from fishing during the closed fishing season between June and August in the YS and ECS. Therefore, the AD and APD are almost zero during this time (Fig 7). After the closed fishing season, AD increased dramatically to a peak of 2.30 million km in November. Similarly, APD showed the same temporal trend with the AD. Except for the closed fishing season, Due to the coming of the Chinese New Year, the AD and APD had another month with low value in February (Fig 7).

## Discussion

The impacts of trawling on the marine benthic environment and biodiversity have been studied intensely over the past two decades [6]. Intensive trawling can alters fish communities by catching unwanted fishes [23]; impacts tourism (e.g., diving) [24]; reduces benthic biodiversity [6]; decreases light levels by resuspension; and damages the seabed by plowing [6]. Recently, large areas of different locations worldwide have banned bottom trawling, e.g., Indonesia, seamounts and hydrothermal vents of New Zealand [24]. Thus, regulating bottom trawling is urgent for protecting the marine environment and biodiversity. This study focused on the impact of bottom trawling on the China Seas, and below we discuss advice and policies for China’s regulation on bottom trawling.

VMS has been proven as an effective tool for monitoring efforts in fisheries [8], and in the future fishery management and conservation on a local scale combing with the logbook data [19, 25]. For fishing grounds in the ECS and YS, VMS has been applied to monitor fishing activities over recent years, e.g. the Xiangshan Port was one of the earliest ports covered VMS, and covered broad range of engine power (Figs 1 and 2). However, the roles of VMS were largely overlooked [15]. For the application of VMS on the fishery managements, a crucial step is to estimate the path width for bottom trawling [18]. In this study, a power function fit between engine power and door width was estimated. Although parameters estimated in our study is different with the previous study in the Europe by Eigaard et al (2015) [18], due to different target species and fishing concept, the fit in this study is suitable for application for Chinese bottom single rig trawler in the YS and ECS. Furthermore, although our study is limited for explicit and accurate estimation on fishing effects for the ECS and YS, patterns of AD, APD, and fishing intensity are acceptable since similar trawling method applied in the fishing grounds in the YS and ECS. (Figs 4 and 5). The fact that patterns among the three indexes have similar distribution in the YS and ECS indicated that these indices can monitor fishing effort. The fishing effort was underestimated because only a minor fraction of vessels could be included in our analysis. The registered single rig otter trawlers in the Xiangshan Port only account for 3.9% of the total trawlers in China, and the landing of these bottom trawlers accounts for 6.05% of the total landing in China (Table 2). The mean trawling intensity in the East China Sea and Yellow Sea was 0.73 year^-1^ in 2013 (Table 2). Compared with other areas, taking results in the Europe as an example, the mean trawling intensity in the four fishing grounds were greater than most areas in the Irish Sea (off Sellafield) and the Dogger Bank (Central North Sea)[26]. Further, communication with shippers suggests that fishing effort in the YS and ECS was expected to stay the same over the year. Although the VMS data were limited for historical records, these discussions with shippers will be helpful to know the historical effort roughly.

**Fig 8:**
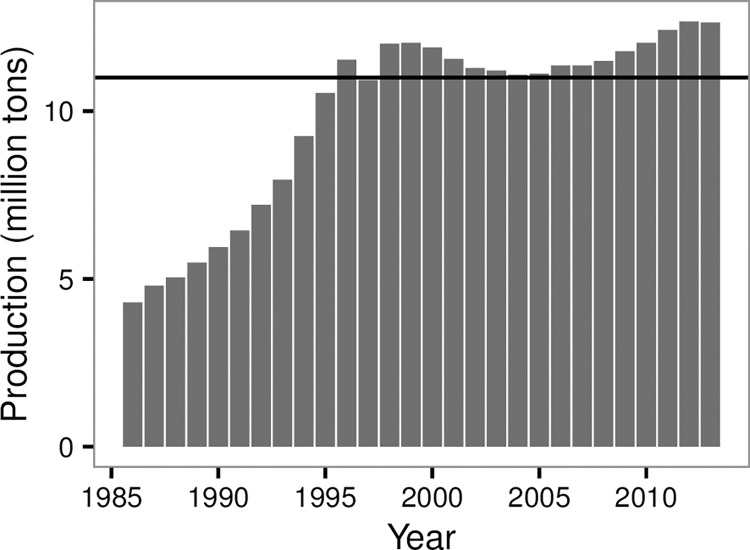
Trends of annual marine fishery production in China Seas between 1986 and 2013. The solid line is the production of 11 million tons.

Overfishing in China has been concerned widely [10], and the marine production each year was more than 11 million tons since 1998 (Fig 8). Fortunately, China has moved towards managing its fisheries. For instance, Hong Kong banned trawling on 31 December, 2012 [27, 28]. The AD and APD measurements indicated two distinct low periods in the YS and ECS. The first one is the closed fishing season between June and September each year; all fishing is forbidden during this time. Thus, the AD and APD were zero during the above time interval (Fig 7). The other low period is around Chinese New Year (usually in February), when Chinese families spend more time together. However, some vessels still worked in this period. Several studies showed bottom trawling can disturb the seabed [3, 29–31], and banning/recovering fishing is best way to protect a fishery [32–34]. Although the fishery management of summer moratorium of marine fishing has been implemented in the YS and ECS since 1995, the annual production of Chinese marine fishery was still more than 11 million tons since 1998 (Fig 8). In addition, fishing grounds near the ports had greater disturbance (Figs 4–6). Currently, the VMS are been introduced on more vessels in China. In this study, we showed the VMS plays effective role on detecting the fishing effort in different fishing grounds in China, and showed the fishing effort patterns in the YS and ECS. Following the pattern, we suggest the different communities (e.g. policy makers, fishery biologists) pay more focuses on areas with higher fishing efforts and make specific fishing polices, e.g. the Yushan fishing ground.

## Supporting Information

S1 File Engine power (kW) and gear width (m) in Chinese trawlers. (XLSX)

S2 File Accumulated distance, accumulated power distance, and fishing intensity in each 0.01° ×0.01° grid cell in the Yellow Sea and East China Sea. (CSV)
